# A review of preparation methods and biomedical applications of micro-nano magnetic particles

**DOI:** 10.1016/j.isci.2026.116924

**Published:** 2026-07-23

**Authors:** Zhijie Huan, Lingtao Meng, Weicheng Ma, Jingjie Zhang, Yiyang Ma, Jiaxin Tu, Weiming Shao, Wei Zhou, Tao Luo

**Affiliations:** 1 School of Electrical Engineering and Automation, Xiamen University of Technology, Xiamen 361024, Fujian, China; 2 Pen-Tung Sah Institute of Micro-Nano Science and Technology, Xiamen University, Xiamen 361102, Fujian, China; 3 Department of Orthopaedic, The 909thHospital, School of Medicine, Xiamen University, Zhangzhou 363000, Fujian, China; 4 Xiamen Key Laboratory of Frontier Electric Power Equipment and Intelligent Control, Xiamen 361024, Fujian, China; 5 Shenzhen Research Institute of Xiamen University, Shenzhen 518000, Guangdong, China

**Keywords:** Materials science, Nanomaterials, Biomedical materials

## Abstract

With the continuous advancement of micro-nano technology, micro-nano magnetic particles have found widespread applications in biomedical fields (including drug delivery, biosensing, and cell therapy) owing to their unique functions and biocompatibility. Their magnetism primarily originates from magnetic materials in the core, including metals, metal alloys, and ferrite-based ceramic materials. Through physical or chemical methods, these magnetic materials are incorporated into biocompatible matrices to form micro-nano magnetic particles with uniform size, excellent stability, and multifunctionality. Properties of these particles tailored by fabrication methods also affect their biomedical applications. This paper reviews the physical and chemical fabrication methods of micro-nano magnetic particles, summarizes their biomedical applications comprehensively, and further discusses the current challenges in their fabrication as well as prospects for their future biomedical applications.

## Graphical abstract

**Figure:**
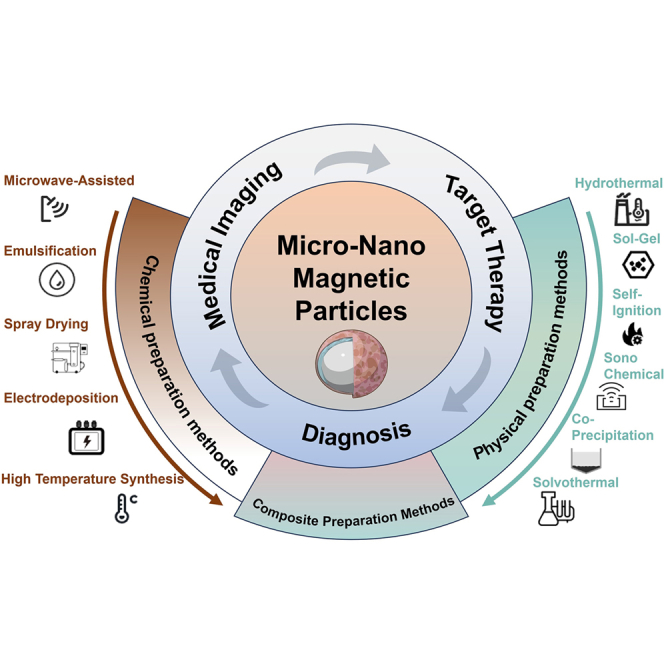

## Introduction

**Figure 1:**
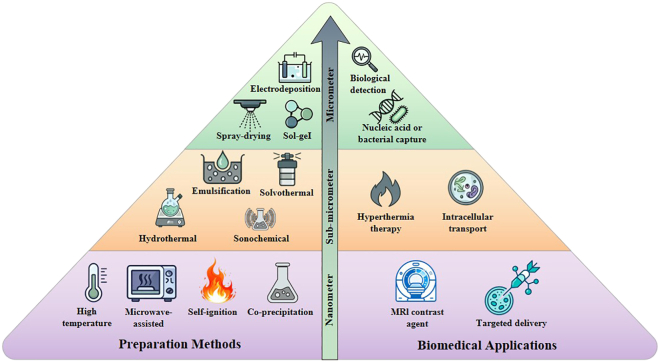
Overview of the preparation and applications of MNMPs

Micro-nano magnetic particles (MNMPs) have demonstrated extensive potential uses across multiple fields due to their special physical and chemical properties, particularly garnering significant attention in biomedical engineering. These particles with micro/nanoscale dimensions not only exhibit intrinsic properties of magnetic materials (e.g., a high specific surface area, superparamagnetism,^1^and excellent dispersibility) but also feature remarkable biocompatibility and biodegradability, but also possess remarkable biocompatibility and biodegradability. These attributes enable their critical roles in key biomedical processes, including cell analysis,^2^ controllable drug delivery,^3^ and cell separation/labeling.^4^ This review systematically summarizes diverse preparation methods of MNMPs and their multifaceted biomedical applications, aiming to provide a comprehensive reference for related research in biomedical engineering. Advances in manufacturing technology have not only refined conventional methods, such as coprecipitation, emulsion templating, sol-gel processing, hydrothermal preparation, and solvothermal routes, but also catalyzed emerging bioinspired preparation methods mimicking natural morphologies via 3D printing.^5^ These preparation methods, subject to different response mechanisms and conditions, enable precise control of particle characteristics, including size, morphology (spherical, rod-like, or dendritic), magnetic saturation, and surface functionality. Such tunability establishes a robust foundation for preparing MNMPs satisfying heterogeneous biomedical requirements. This methodology versatility ensures the selection of optimal preparation methods to obtain customized MNMPs for various biomedical applications, spanning contrast-enhanced imaging,^6^ therapy platforms,^7^ and other emerging biomedical fields.^8^ In the last decade, MNMPs have been widely studied for biomedical applications. In disease diagnosis, they are used as magnetic resonance imaging (MRI) contrast agents for image quality enhancement,^9^ facilitating early diagnosis and accurate localization. Especially when modified with certain ligands, they can specifically bind to tumor cells, clearly outlining tumor contours. In addition, these particles can be used for magnetic separation and enrichment of specific cells or molecules in biological samples, improving detection efficiency and sensitivity. In drug delivery, MNMPs could be actuated by external magnetic fields to achieve precise drug delivery and targeted release, enhancing therapeutic effects and reducing side effects, particularly showing great potential in cancer treatment.^10^ During cell manipulation, MNMPs can bind to cell surface receptors or antigens, marking and isolating target cells, promoting cell biology research and the development of regenerative medicine,^11^ such as guiding stem cells to migrate to damaged tissues for repair.^12^ Thus, this paper reviews the fabrication processes of MNMPs and their state-of-the-art applications in the biomedical field. Firstly, two major categories of preparation methods, physical methods and chemical methods, are provided. The specific steps and processes of various preparation methods are described in detail. Secondly, the application of MNMPs in the biomedical field is elaborated, as well as their versatility and development potential. Finally, the future development of MNMPs is prospected and summarized (Figure 1).

## Preparation of MNMPs

The preparation of MNMPs is an integrated process combining chemical preparation, materials science, and nanotechnology, requiring precise control over particle size, morphology, chemical composition, and magnetic properties.^13^ They are typically made of magnetic metals such as iron, cobalt, nickel, or their oxides through sophisticated chemical processes, including one-pot preparation,^14^ two-step methods,^15^ multi-step approaches,^16^ and *in situ* reduction techniques.^17^ Recently, researchers have improved the traditional methods to prepare functional MNMPs. For example, FePt nanopropellers have been fabricated via the oblique angle deposition technique (a physical vapor deposition method),^18^ while CoNi magnetic alloy/graphitized carbon composite spherical microparticles (for electromagnetic pollution mitigation) have been synthesized via solvothermal methods coupled with annealing in an inert atmosphere.^19^ These methods have enabled the regulation of physicochemical properties and the enhancement of particle stability and functionality through surface modification and coating techniques. Based on differences in preparation principles and technical routes, various methods exhibit distinct characteristics in the control of reaction conditions, regulation of particle morphology and magnetic properties, as well as adaptability to large-scale production. The following sections will elaborate on the specific processes, core parameters, and preparation advantages of each method.

### Physical preparation methods

As an important technical type for the controllable fabrication of MNMPs, physical preparation methods rely on the regulation of physical fields, mechanical effects, or electrochemical reactions, and realize the shaping and assembly of magnetic particles by adjusting key physical parameters such as electric field, temperature, and mechanical force. This category of methods is characterized by direct preparation processes and good production scalability, with typical technical routes including electrodeposition, emulsification, and high-temperature synthesis, each possessing distinctive advantages in the regulation of particle structural order and morphology, as illustrated in Table 1.

**Table 1:** Comparison of physical preparation methods for preparing magnetic particles

Method	Size coercivity	Saturation magnetization	Coercivity	Cost	Reference
Electrodeposition	50 nm to 5 μm	150 to 220 emu/g	80 to 400 Oe	medium cost	29
Emulsification	300 nm to 1 mm	8 to 65 emu/g	1 to 20 Oe	low cost	33–35
High temperature	1 nm to 300 μm	20 to 100 emu/g	0 to 100 Oe	high cost	47,48

#### Electrodeposition method

**Figure 2:**
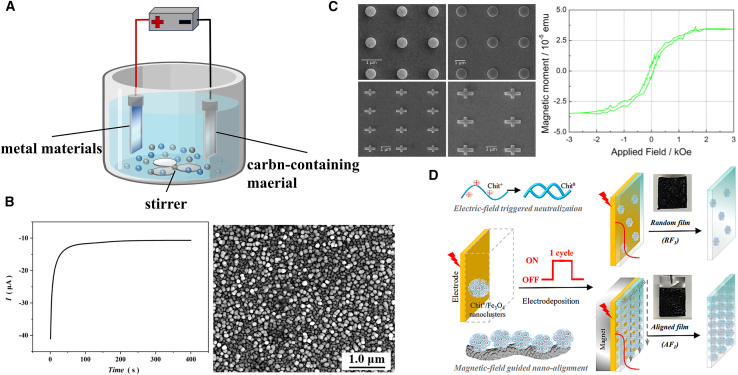
Electrodeposition method for preparing MNMPs (A) Schematic diagram of magnetic particle preparation via the electrodeposition method. (B) Electrodeposition preparation curve and TEM image of MNMPs.^21^ (copyright 2021, Elsevier). (C) AFM images and hysteresis loops of MNMPs.^22^ (copyright 2020, Elsevier). (D) Schematic illustration of the electrodeposition process.^23^ (copyright 2023, Elsevier).

The electrodeposition method relies on electrochemical processes. With the applied electric field in an electrolyte solution containing metal ions (such as *Fe*^2+^, *Co*^2+^, *Ni*^2+^, and so forth), metal ions are reduced to gain electrons at the cathode surface, forming deposited MNMPs,^20^ as illustrated in Figure 2A. The electrodeposition method enables precise regulation of parameters such as current density, deposition time, electrolyte composition, and temperature, allowing the preparation of MNMPs ranging from micro to nano scale. This method also facilitates precise morphological control and could produce particles with custom shapes, including spherical, rod-like, and cubic structures.^24^ Thus, electrodeposition is highly adaptable to those applications requiring specific structural features in MNMPs.

The electrodeposition method has been widely used to prepare MNMPs with uniform size, high activity, and biocompatibility. As shown in Figure 2B, the deposition process on the electrode surface occurs rapidly during preparation, followed by the stabilization of the current response. The prepared MNMPs exhibit a uniform diameter of 2.8 *μ*m with a slightly rough surface, which enhances their specific surface area to improve adsorption capacity and biocompatibility. Also, electrodeposition could prepare well-dispersed MNMPs without significant agglomeration, which is a critical feature for optimizing their performance in biosensing applications.^21^ Figure 2C demonstrates that electrodeposition allows precise control of magnetic particle growth, resulting in uniformly sized particles with different shapes without overgrowth or uneven distribution. Hysteresis loop characterization reveals a coercivity of 81 Oe, and it tends to saturate when the applied magnetic field reaches about 400 Oe, presenting typical ferromagnetic characteristics.^22^ Moreover, since this method is conducted under normal environmental conditions, the preparation process could avoid energy-intensive high-temperature or high-pressure conditions, thereby reducing energy consumption and environmental impact. As illustrated in Figure 2D, magnetic composite particles were fabricated by combining electrodeposition with an external magnetic field. Within the magnetic field, combined with oscillating electrical signals, chitosan and *Fe*_3_*O*_4_ nanoclusters were self-assembled into oriented structure hydrogels by electrodeposition, forming highly ordered “fiber-like” aligned structures. Then, an electric field is utilized to trigger water decomposition, generating a high pH that triggers the local gelation of chitosan. Meanwhile, the introduced magnetic field has enhanced the enrichment of particles on the electrode surface and increased magnetic content in the composite film significantly. This technique highlights the synergistic capability of electrodeposition and magnetic fields to precisely regulate the structure and properties of composite materials.^23^

However, the electrodeposition method faces purity control challenges. Impurity ions present in the electrolyte are prone to being incorporated into the products during the deposition process, which impairs the performance stability of the MNMPs. In addition, this method imposes extremely high requirements on the precision of reaction parameter regulation; improper operation can easily result in uneven particle morphology. Furthermore, its deposition efficiency for certain magnetic materials with high melting points is relatively low, making it difficult to achieve efficient preparation.

#### Emulsification method

**Figure 3:**
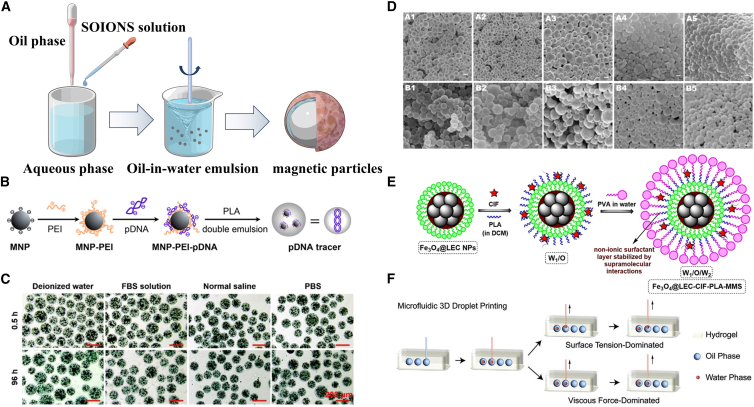
Emulsification method for preparing MNMPs (A) Schematic diagram of the preparation of MNMPs via the emulsification method. (B) Preparation scheme of the pDNA-based tracking system.^26^ (copyright 2020, Elsevier). (C) Image of the stability test results for magnetic microspheres.^27^ (copyright 2022, Elsevier). (D) SEM images of MNMPs with different ratios.^28^ (copyright 2016, Elsevier). (E) Process diagram of the preparation of MNMPs using the double emulsification method.^29^ (copyright 2023, Elsevier). (F) Schematic diagram of microfluidic 3D droplet printing within droplets.^30^ (copyright 2021, Wiley).

The emulsification method utilizes the property of two immiscible liquids forming a stable emulsion system under the action of surfactants. In this method, droplets primarily serve as microreactors. During preparation, an aqueous phase containing magnetic material precursors is mixed with an oil phase containing surfactants and organic solvents. Through mechanical stirring or ultrasonic treatment, the aqueous phase is dispersed into tiny droplets that become uniformly distributed within the oil phase, forming a water-in-oil (W/O) emulsion.^25^ These miniature aqueous droplets act as individual microreactors, providing isolated spaces for subsequent chemical reactions, as illustrated in Figure 3A.

The emulsification method is usually employed to prepare functional MNMPs, typically consisting of a magnetic core and a functional shell. As illustrated in Figure 3B, this approach effectively combines MNMPs with plasmid DNA, which are both encapsulated within polylactic acid microspheres in the size of 200–300 *μ*m. Moreover, MNMPs were modified with polyethyleneimine to make them positively charged and combine with negatively charged plasmid DNA. Then, they were encapsulated in polylactic acid microspheres by the double emulsification method, and the dispersibility was improved by charge repulsion and steric hindrance. This encapsulation not only protects plasmid DNA from degradation in complex aqueous environments, but also leverages the buoyancy properties of polylactic acid microspheres for efficient surface concentration and collection, thereby enhancing tracking control efficiency.^26^ As shown in Figure 3C, MNMPs prepared by emulsification in the size of 79.8 *μ*m–362.3 *μ*m maintained a clear spherical shape in deionized water, FBS solution, normal saline and PBS, without significant deformation or aggregation.^27^ SEM images shown in Figure 3D indicate that these particles prepared by emulsification display in spherical or nearly spherical shape. Although slight size variations were observed with increasing loading ratios of iron oxide nanocrystals (NCs), the overall morphology remained stable, with the size between 300 and 600 nm.^28^

What’s more, the double emulsion method is an advanced technique derived from traditional emulsification methods for preparing magnetic microspheres. It involves two steps of the emulsification process. Firstly, an aqueous phase containing magnetic material precursors is dispersed in an oil phase containing surfactants and organic solvents, forming a primary W/O emulsion. Subsequently, the W/O emulsion is further dispersed into an external aqueous phase containing different surfactants, forming a water-in-oil-in-water (W/O/W) double emulsion, as illustrated in Figure 3E. Through double emulsification process, drugs and MNMPs could be dispersed within the polymer matrix uniformly with a core-shell structure.^29^ Microfluidic technology also provides an ideal microreactor for the preparation of MNMPs by emulsification through the customized microchannel structure and controllable fluid flow rate in microfluidic chip.^31^ In contrast to traditional emulsification methods, it enables efficient mixing at low flow rates while providing superior control over droplet size and generation frequency, thereby improving the dimensional uniformity and stability of MNMPs. Additionally, this technique could minimize the usage of reagent consumption, surfactants, and organic solvents, and effectively reduce the costs and environmental impact. As shown in Figure 3F, the combination of microfluidics with 3D printing technology allows precise printing of oil droplets within hydrogel matrices, followed by controlled deposition of aqueous cores inside oil droplets, forming W/O/W double emulsion structures.^30^ The microfluidic-assisted 3D droplet printing technique provides a novel method for simpler and more flexible preparation of functional MNMPs with promising applications in biomedical detection and related fields. However, due to the limitations of 3D printers’ resolution and nozzle diameter, the diameter of the emulsion that can be prepared is between 500 and 3000 *μ*m. Moreover, the efficiency of preparing double emulsions is approximately 300 per hour, so parallel printing with multiple nozzles is needed to enhance large-scale production capacity.^32^

However, the emulsification method involves a complex and time-consuming operation process that requires strict control of the oil-water phase ratio, surfactant concentration, and stirring conditions, thus posing major challenges for large-scale industrial fabrication. The uniformity of particle size prepared by this method is susceptible to the stability of the emulsion. Additionally, subsequent removal of the organic phase and surfactants is necessary, and residual impurities may reduce the biocompatibility or magnetic properties of the products, thereby increasing the post-treatment costs.

#### High temperature synthesis method

**Figure 4:**
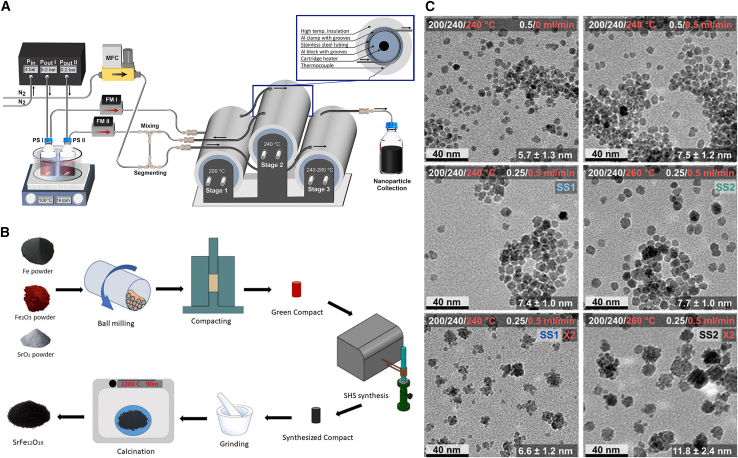
High temperature synthesis method for preparing MNMPs (A) Schematic diagram of the high temperature synthesis setup.^34^ (copyright 2023, Elsevier). (B) Schematic illustration of the self-propagating high temperature synthesis (SHS) method for preparing MNMPs.^35^ (copyright 2024, Elsevier). (C) TEM image of iron oxide nanoparticles (IONPs) prepared by high temperature synthesis.^34^ (copyright 2023, Elsevier).

The high temperature synthesis method is realized by breaking down metal precursors into magnetic crystal nuclei in an inert gas environment, such as metallic elements, metal oxides, ferrite powders or rare earth permanent magnetic alloys and other substances with high thermal stability and magnetic responsiveness, where the dynamic regulation of nucleation and growth kinetics is achieved through the addition of surfactants.^33^ The schematic diagram of the high temperature synthesis setup is shown in Figure 4A^34^ By regulating the temperature distribution through a three-stage high-temperature flow reactor and combining it with the self-seed growth strategy, the prepared iron oxide nanoparticles form a single magnetite/magneto hematite phase with high crystallinity. Moreover, the nucleation and growth processes are optimized through segmented temperature control, thereby possessing good monodispersity and strong magnetic responsiveness. Moreover, the self-propagating high temperature synthesis (SHS) method is a derived technique from the conventional high temperature synthesis method, which introduces a self-sustaining exothermic chain reaction mechanism in the reactant system. By controlling steps such as raw material mixing, ball milling,^35^^,^^36^ compacting, reaction conditions, and post processing, SHS method can be utilized to prepare MNMPs with excellent properties.^37^ Compared to traditional methods that rely on continuous external heating, SHS method offers advantages such as shorter reaction time, lower energy consumption and higher product purity, which is particularly suitable for preparing submicron scale MNMPs, with an average size of 325 nm, as depicted in Figure 4B. These methods could enhance particle dispersion and biocompatibility through surface functionalization and have been broadly utilized in magnetic hyperthermia therapy, high-frequency magnetic cores, and targeted drug delivery.^38^

MNMPs prepared by high-temperature synthesis methods usually have high purity and well-crystallized structures,^39^ since the elevated temperature conditions help to eliminate impurities and defects. Higher reaction temperatures and prolonged reaction durations may promote particle growth, yielding larger-sized MNMPs, while specific temperature gradients and atmospheric conditions can produce MNMPs with unique morphologies.^40^ As shown in Figure 4C, the prepared MNMPs exhibit relatively uniform size distribution, a multi-core structure, and excellent crystallinity (the size range is from 2 to 17 nm).^34^

However, the high-temperature synthesis method relies on specialized high-temperature equipment. Not only are the procurement and maintenance costs of the equipment high, but it also consumes a substantial amount of energy, which limits its large-scale industrial application. The reaction process of this method is complex, imposing extremely stringent requirements on the control of conditions such as temperature gradients and inert gas atmospheres. Meanwhile, particles tend to grow excessively large in a high-temperature environment, making it difficult to precisely regulate the size of nano-scale products. Additionally, the subsequent cooling process may induce particle agglomeration.

### Chemical preparation methods

Chemical synthesis methods are one of the most widely used technical systems for preparing MNMPs, which rely on hydrolysis, polycondensation, precipitation, and redox reactions of magnetic metal precursors in solution or closed reaction systems to drive the nucleation and growth of magnetic particles. By precisely regulating the precursor ratio, pH value, reaction temperature and other key conditions, this type of method enables fine control of the crystal structure, magnetic properties and surface functionality of MNMPs, and the mainstream representative methods include sol-gel, hydrothermal, self-ignition, solvothermal and co-precipitation methods, as illustrated in Table 2.

**Table 2:** Comparison of chemical preparation methods for preparing magnetic particles

Method	Size coercivity	Saturation magnetization	Coercivity	Cost	Reference
Sol-gel	30 nm–100 nm	8 to 80 emu/g	40 to 1000 Oe	high cost	56–59
Hydrothermal	20 nm–200 nm	30 to 200 emu/g	0 to 100 Oe	high cost	64–66
Self-ignition	10 nm–50 nm	50 to 100 emu/g	0 to 60 Oe	low cost	72,73
Solvothermal	20 nm–400 nm	2 to 150 emu/g	5 to 150 Oe	high cost	78–81
Co-precipitation	5 nm–600 nm	1 to 70 emu/g	0 to 600 Oe	low cost	89–92

#### Sol-gel method

**Figure 5:**
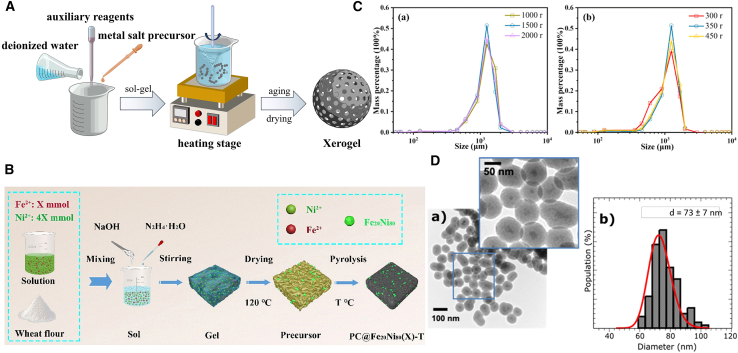
Sol-gel method for preparing MNMPs (A) Schematic diagram of the preparation of MNMPs via the sol-gel method. (B) Process flow for the preparation of MNMPs using the sol-gel method.^43^ (copyright 2023, Elsevier). (C) Size distribution of MNMPs obtained under different shear rates and stirring speeds.^44^ (copyright 2021, Elsevier). (D) TEM image and particle size distribution of MNMPs.^45^ (copyright 2020, ACS).

The sol-gel method has been widely used for the preparation of MNMPs,^41^ as a wet chemical preparation technique. It is based on the hydrolysis and polycondensation of metal alcohol oxides or metal inorganic salts. Taking metal alcohol oxides as an example, they first hydrolyze into metal hydroxide compounds in alcohol solvents, which subsequently undergo polycondensation to form a sol with a three-dimensional network structure. When the magnetic ions are fixed, this sol gradually transforms into a gel. After removing impurities with drying and heating treatment, specific MNMPs could be obtained,^42^ as illustrated in Figure 5A. During the preparation process, the selected precursors are dissolved in a solvent along with additives such as catalysts and surfactants. Under controlled temperature and agitation, hydrolysis and polycondensation of the precursors are promoted, driving the sol-gel transition. Finally, the dried gel (xerogel) is converted to the target MNMPs by heat treatment through phase transition,^43^ as shown in Figure 5B. The prepared MNMPs are crystalline and uniformly dispersed in the porous carbon matrix, with a sub-micron size.

During this preparation process, precursors can be thoroughly mixed in the solution state, ensuring excellent chemical homogeneity.^46^ In addition, precise controlled reaction condition enables accurate regulation of particle size and morphology, providing customized MNMPs for different applications.^47^ As shown in Figure 5C, stirring rate acts as a critical factor affecting the dimension of particles during sol-gel preparation.^44^ Experimental results demonstrate that, at a stirring rate of 350 rpm, the prepared particles exhibit a more concentrated size distribution with no significant spatter during stirring process. The final size of the MNMPs after calcination ranged from 6.03 to 138.72 nm. Concurrently, their saturation magnetization increased from 35.76 to 76.61 emu/g, and the coercivity rose from 89.15 to 928.86 Oe. This enhancement in magnetic properties is attributed to the increased crystallinity resulting from the particle size enlargement. Figure 5D illustrates the morphology and size distribution of multi-core-shell nanoparticles.^45^ Characteristic multi-core-shell architectures of the spherical nanoparticles could be observed from TEM images (the average size is around 73 nm). Due to the excellent colloidal stability of MNMPs during the sol-gel processing, some nanoparticles exist as individual encapsulated particles while others form small aggregates.

However, the sol-gel method requires the use of large amounts of organic solvents during the preparation process. This not only increases production costs but also poses a risk of environmental pollutant emissions, and solvent residues may affect the performance stability of MNMPs. During the drying stage, solvent evaporation can cause shrinkage and cracking of the gel network, damaging the structural integrity and uniformity of the products, which necessitates additional optimization of the drying process. In addition, this method has a relatively long reaction cycle and low preparation efficiency, making it difficult to meet the demands for rapid large-scale production.

#### Hydrothermal method

**Figure 6:**
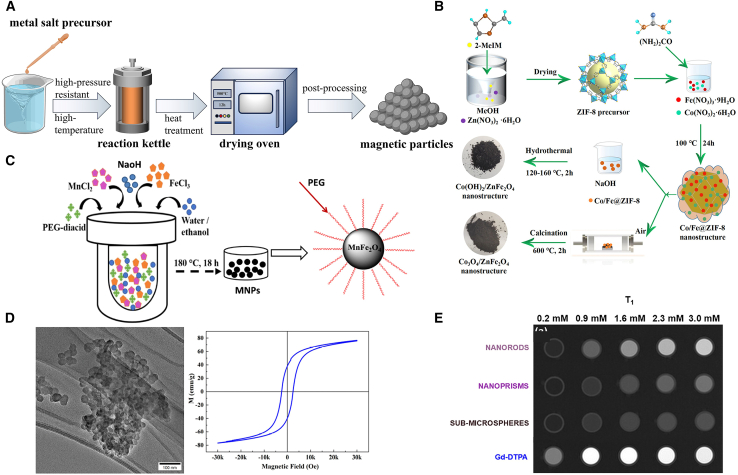
Hydrothermal method for preparing MNMPs (A) Schematic diagram of magnetic particle preparation via hydrothermal method. (B) Process flow for preparing MNMPs through hydrothermal method.^49^ (copyright 2023, Elsevier). (C) Schematic illustration of magnetic nanoparticle preparation via one-pot hydrothermal preparation method.^50^ (copyright 2022, Elsevier). (D) TEM image of MNMPs.^51^ (copyright 2023, MDPI). (E) MRI grayscale image of MNMPs^52^ (copyright 2024, Royal Society of Chemistry).

The hydrothermal method is utilized to prepare MNMPs in a high-temperature and high-pressure aqueous environment, based on the unique physicochemical properties of water. With the increase of the ion product constant and degree of ionization, the solubility and reactivity of reaction substances could be significantly enhanced.^48^ During the preparation of MNMPs, compounds containing magnetic elements, such as a mixed solution of metal ions required for ferrite formation or soluble salts of metals such as iron, cobalt, and nickel, are mixed with a suitable solvent. This mixture is then placed in a dedicated sealed reactor, as illustrated in Figure 6A. Under high temperature and high-pressure conditions, the raw materials undergo a series of chemical reactions in the solution, including dissolution, hydrolysis, and precipitation. By controlling the reaction parameters, the magnetic material gradually crystallizes and finally forms MNMPs with specific crystal structures.^49^ The zinc source was mixed with ferric nitrate and cobalt nitrate solutions. After adding urea, the composite material was prepared through a hydrothermal reaction at 100°C. Then it was mixed with NaOH solution and subjected to a hydrothermal reaction at 120°C–160°C. Finally, it was washed and dried, as shown in Figure 6B. The one-pot hydrothermal preparation method is another efficient preparation method developed from hydrothermal methods.^50^ As illustrated in Figure 6C, in this approach, all reaction raw materials, including solvents, are mixed into one single reaction vessel together. During this process, solutions like NaOH may be added to adjust the pH value accordingly. Subsequently, the reaction system is sealed in a high-pressure autoclave for complex chemical reactions. The prepared MNMPs demonstrate excellent static and dynamic magnetic properties, with a saturation magnetization intensity of 68 emu/g, a coercivity of 30 Oe, and an average particle size of approximately 40 nm.

The hydrothermal method offers several notable advantages for preparing MNMPs. As shown in Figure 6D, it achieves controllable crystal structure and morphology for MNMPs under relatively mild conditions.^53^ By adjusting parameters such as reaction temperature, time, pH of solution, and additives, MNMPs with spherical, rod-like, or cubic morphologies and uniform size distribution can be synthesized, without an obvious hysteresis phenomenon.^51^ Moreover, the prepared MNMPs exhibit high crystallinity and excellent purity, as illustrated in Figure 6E. In the high-temperature and high-pressure aqueous solutions, impurity ions are not inclined to participate in the reaction, facilitating the formation of pure magnetic crystal structures for enhancing the magnetic properties.^52^ Hydrothermally prepared MNMPs demonstrate significant value for multiple practical applications owing to their superior properties. In the biomedical field, they can be used to prepare MRI contrast agents and targeted drug delivery carriers.^54^ With excellent magnetic properties and controllable sizes, these particles enable precise detection and treatment of disease.^55^ For environmental governance, they could serve as magnetic adsorbents to remove heavy metal ions and organic pollutants from water bodies, thereby achieving environmental purification and restoration.^56^

However, the hydrothermal method requires the use of specialized high-temperature and high-pressure reactors as well as supporting temperature and pressure monitoring systems, which leads to high equipment costs and certain safety risks associated with the high-pressure environment. The reaction parameters of this method exert a significant influence on product performance and are rather difficult to regulate, which easily results in problems such as uneven particle morphology or insufficient crystallinity. Meanwhile, the closed nature of the reaction system makes real-time monitoring of the reaction process difficult, which is unfavorable for process optimization and *in situ* process control.

#### Self-ignition method

**Figure 7:**
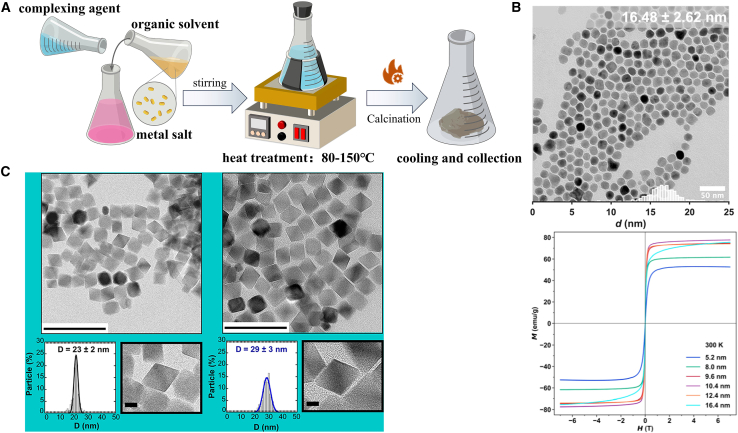
Self-ignition method for preparing MNMPs (A) Schematic diagram of magnetic nanoparticle preparation via self-ignition method. (B) Size and size distribution of MNMPs in different samples and the magnetization curve of MNMPs.^59^ (copyright 2022, ACS). (C) TEM images of MNMPs.^60^ (copyright 2021, ACS).

The self-ignition method, also known as SHS, is a highly distinctive approach for preparing MNMPs.^57^ Based on the substantial heat released by the chemical reaction itself, it could promote the reaction to advance rapidly and propagate continuously within an extremely short time, the same process as combustion. Suitable reactants for this method usually consist of compounds containing magnetic elements, such as combinations of metal oxides, and reducing agents, like an iron oxide and aluminum powder mixture. The reactants should be thoroughly homogenized according to a precise stoichiometric ratio to ensure completeness and uniformity in subsequent reactions. After providing an initial energy input to a localized area by ignition, the reaction rapidly commences. The initial reaction zone releases intense heat, sufficient to ignite adjacent regions of reactants, allowing wave-like propagation of the reaction throughout the entire system,^58^ as illustrated in Figure 7A.

During this intense reaction process, the transient high temperature triggers a complex series of chemical reactions between reactants, including redox and combination reactions. These reactions promote the formation of magnetic substances, and their crystallization and agglomeration to generate MNMPs ultimately. The whole reaction usually happens within a few seconds to tens of seconds. As shown in the TEM images in Figure 7B, the prepared nanoparticles exhibit excellent monodispersity, whose size can be precisely controlled between 4.24 and 16.48 nm by preparation conditions.^59^ Its superior size control capability enables continuous size modulation in different dimensions with favorable uniformity. At room temperature, the magnetization curve has no hysteresis loop and shows typical superparamagnetic behavior. Figure 7C illustrates that this method achieves both precise size control and shape regulation of nanoparticles, whose size is adjustable between 20 and 40 nm.^60^ In addition, since the rapid reaction process minimizes impurity incorporation and crystal defect formation, this method could prepare MNMPs with high purity and well-defined crystallinity. In the biomedical field, these prepared MNMPs could be utilized as drug carriers and MRI contrast agents^61^ and enable precise detection and treatment^62^ of pathological sites.

However, the self-ignition method involves an instantaneous high temperature and intense exothermic process during the reaction, which poses considerable safety risks and imposes extremely high requirements on the high temperature and impact resistance of the reaction vessel. The reaction rate of this method is excessively fast, making it difficult to precisely control the size and morphology of the products and easily leading to particle agglomeration or crystalline defects.

#### Solvothermal method

**Figure 8:**
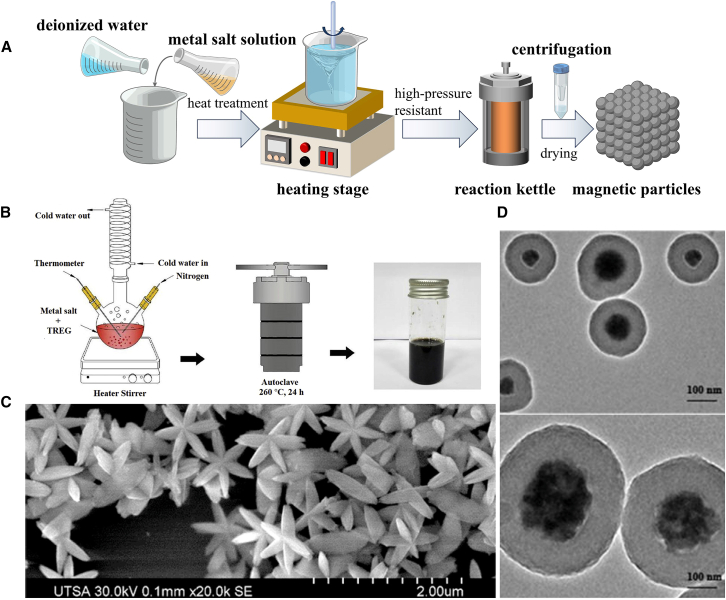
Solvothermal method for preparing MNMPs (A) Schematic diagram of magnetic particle preparation via the solvothermal method. (B) Schematic illustration of magnetic nanoparticle preparation by the solvothermal method.^64^ (copyright 2020, Elsevier). (C) SEM image of magnetic microparticles.^65^ (copyright 2016, Elsevier). (D) TEM images of magnetic microspheres after different temperature treatments.^66^ (copyright 2020, Elsevier).

The solvothermal method introduces high temperatures and pressures in solvents to promote chemical reactions. The materials used are usually organic or inorganic salts of metals, such as iron, cobalt, and nickel, including iron acetylacetonate, nickel chloride, and other metal oxide precursors or metal ion compounds that can react in non-aqueous solvents to form magnetic phases. Organic solvents are utilized as the reaction medium instead of water in a sealed reaction system, whose unique physicochemical properties under elevated temperature and pressure could facilitate chemical reactions,^63^ as illustrated in Figure 8A. During the preparation, certain compounds containing magnetic elements, such as metal salts or metal organic complexes, are selected as the raw materials, which are dissolved uniformly in specific organic solvents (e.g., ethanol, ethylene glycol, dimethylformamide) in controlled ratios. The organic solvent not only serves as the reaction medium but may also participate in the reaction, enhancing the growth and properties of the MNMPs. The uniform mixture is then transferred into a specialized high-pressure sealed autoclave for heating. Under high temperature and high-pressure conditions, the dissolving capacity and reactivity of the solvent are significantly improved, triggering a series of complex chemical reactions (e.g., hydrolysis, reduction, coordination),^64^ as shown in Figure 8B. These reactions promote the nucleation and growth of magnetic materials, ultimately yielding particles with tailored crystal structures and proper magnetic properties, with a saturation magnetization intensity of 58.8 emu/g and an average size of 18.5 nm.^67^

With the solvothermal method, size and morphology of particles could be controlled precisely by regulating reaction conditions, and dispersibility and stability could be enhanced with surface modification through the addition of surfactants or ligands.^68^ As demonstrated in Figure 8C, submicron star-shaped MNMPs could be prepared, with a distinctive six-petaled star-like structure and approximately 600 nm in length. After heat treatment at 800°C, these particles would transform into isolated petal-like configurations.^65^ The six-petaled structures with specific crystallographic orientations are formed by the gradual crystallization and growth of reactants under solvothermal conditions. Within a certain temperature range during the solvothermal method, the microspheres maintained uniform core-shell architecture and monodisperse spherical morphology. TEM images in Figure 8D clearly illustrate the morphological evolution of core-shell MNMPs with carbonization. The particle diameter and carbon shell thickness closely resemble those of the original MNMPs, without significant deformation or collapse. Moreover, after carbonization and sulfonation treatment, it can still maintain a uniform spherical morphology and good monodispersicity, without an obvious agglomeration phenomenon. This indicates superior thermal stability of the carbon shells within a specific temperature range.^66^ The solvothermal method offers advantages including excellent crystallinity, controllable surface modification capabilities, and exceptional stability. In addition, featuring superparamagnetic properties and a saturation magnetization of 10.6 emu/g, the prepared MNMPs are highly suitable for biomedical applications.^69^^,^^70^

However, the organic solvents employed in the solvothermal method are mostly flammable, explosive, and volatile, which imposes higher requirements on experimental operation and equipment protection, and entails high costs for solvent recovery and treatment. The reaction system of this method is non-aqueous, leading to great difficulties in the post-treatment of products, and residual solvents may impair their biocompatibility. Meanwhile, the selection of solvents exerts a significant influence on the structure and performance of products, and a large number of experiments are required to screen suitable solvents, which increases the research and development costs.

#### Co-precipitation method

**Figure 9:**
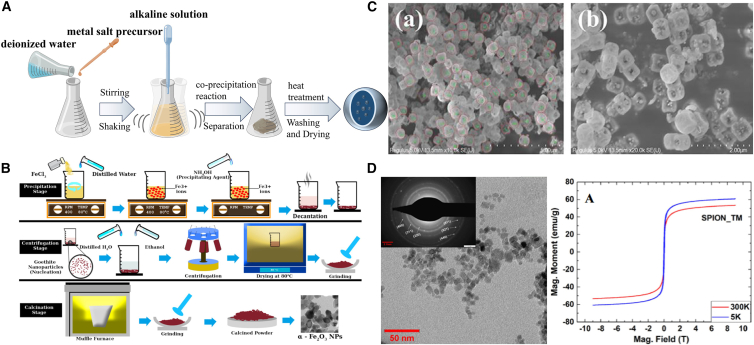
Co-precipitation method for preparing MNMPs (A) Schematic diagram of preparing MNMPs via the co-precipitation method. (B) Preparation steps for synthesizing MNMPs by co-precipitation method.^72^ (copyright 2023, Elsevier). (C) SEM image of MNMPs.^73^ (copyright 2022, Elsevier). (D) TEM image of MNMPs.^74^ (copyright 2020, Elsevier).

The co-precipitation method is another typical technique for rapid preparation of MNMPs through liquid phase reactions. During the process, metal salt solutions containing magnetic metal precursors elements and precipitating agents are mixed to yield hydroxides or salt precipitates containing multiple metallic elements by simultaneous precipitation reactions of the metal cations.^71^ As illustrated in Figure 9A, with precise control of solution pH, temperature, metal ion concentration, and precipitating agent addition rate, the magnetic materials are precipitated with specific crystal structures and chemical compositions, forming MNMPs with tailored magnetic properties.^75^ In biomedical applications, these particles are commonly employed as MRI contrast agents to enhance disease diagnosis clarity,^76^ and targeted drug carriers that deliver therapeutics precisely to lesion sites under external magnetic fields for efficient treatment.^77^ In addition, they find use in cell separation and magnetic hyperthermia therapies.^78^

The preparation process of the co-precipitation method is relatively simple. Firstly, metal salts, such as ferrous sulfate and ferric chloride, are accurately weighed and dissolved in deionized water in specific proportions to form a uniform mixture. Then, as the precipitant is dripped into the solution under vigorous stirring, the metal cations in the solution rapidly react with the anions from the precipitant. During the reaction, the temperature is generally maintained between 60°C and 90°C with the pH value kept at 8–11 to ensure smooth reaction progress and product quality. After precipitation, MNMPs are separated from the solution by centrifugation or filtration and washed over with copious amounts of deionized water to remove surface-adsorbed impurity ions and residual precipitant. Finally, the washed samples are dried at 60°C–80°C. In order to further optimize magnetic properties, post-treatments such as annealing are conducted accordingly,^72^ as illustrated in Figure 9B. The entire process demonstrates low energy consumption, with relatively low prices of raw materials, which could effectively reduce preparation costs. Through precise regulation of reaction conditions, this method enables effective size control for MNMP preparation,^79^ while ensuring uniform chemical composition and stable product quality with an average size of approximately 610 nm,^78^ as illustrated in Figure 9C. The co-precipitation method also shows great potential for controlling the morphology of MNMPs. As shown in Figure 9D, nanoparticles prepared through two distinct energy sources (thermal energy and microwave energy) exhibit rectangular and spherical shapes, respectively, with excellent single-crystal characteristics. Moreover, they are Superparamagnetic Iron Oxide Nanoparticles (SPIONs) with a size of 6–7 nm and a saturation magnetization of approximately 53 emu/g. After being coated with serine, they can efficiently load doxorubicin and exhibit drug delivery capabilities in pH-responsive release and endocytosis experiments.^74^

However, MNMPs fabricated via the co-precipitation method show poor surface charge stability and tend to agglomerate due to interparticle attractive forces, which degrade their dispersibility and application performance. This method is extremely sensitive to variations in reaction conditions, and minor fluctuations may lead to an inhomogeneous chemical composition of the products, thus reducing the consistency of their performance.^80^ In addition, impurity ions are easily introduced during the precipitation process, which necessitates repeated washing and purification steps and consequently increases the post-treatment procedures and costs.

### Composite preparation methods

Composite preparation methods for MNMPs are innovative technical strategies formed by integrating the advantages of single physical or chemical preparation methods and introducing exogenous auxiliary fields or process technologies, which aim to make up for the shortcomings of single methods in preparation efficiency, particle dispersibility and performance regulation. By combining microwave, spray drying, sonochemical and other auxiliary technologies with traditional preparation processes, this category of methods can effectively optimize the nucleation and growth process of magnetic particles, and the prepared MNMPs exhibit more excellent comprehensive properties to adapt to diverse biomedical application requirements, as illustrated in Table 3.

**Table 3:** Comparison of composite preparation methods for preparing magnetic particles

Method	Size coercivity	Saturation magnetization	Coercivity	Cost	Reference
Microwave-assisted	20 nm–300 nm	15 to 80 emu/g	4 to 20 Oe	high cost	20–25
Spray drying	1 μm–100 μm	40 to 90 emu/g	0 to 10 Oe	high cost	41–45
Sono chemical	10 nm–100 nm	3 to 90 emu/g	0 to 10 Oe	high cost	96–99

#### Microwave-assisted method

**Figure 10:**
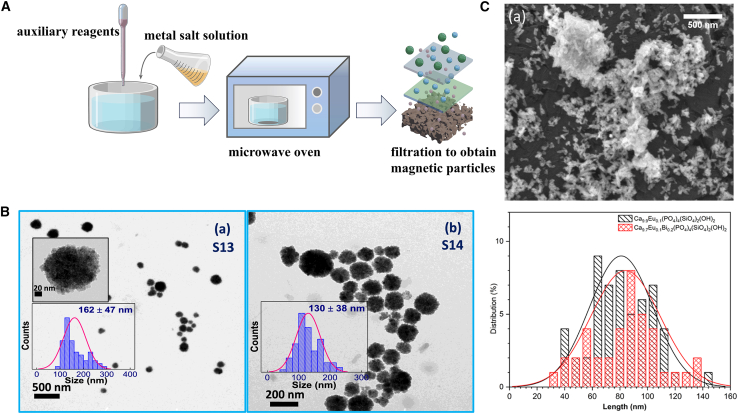
Microwave-assisted method for preparing MNMPs (A) Schematic diagram of the microwave-assisted method for preparing MNMPs. (B) TEM images of different MNMPs showing their morphology and size distribution.^81^ (copyright 2021, Elsevier). (C) SEM images of MNMPs and their corresponding size distribution chart.^82^ (copyright 2020, ACS).

The microwave-assisted method relies on the rapid heating characteristics of microwaves, where high-frequency electromagnetic waves interact with molecules in the reaction system, inducing rapid molecular vibration and rotation to generate an internal heating effect. The prepared materials are usually iron-based oxides, cobalt-based/nickel-based materials, ferrites, and magnetic polymer composites, which have good magnetic responsiveness and can effectively absorb microwave energy. This accelerates the reaction process, as illustrated in Figure 10A.

The rapid heating ability of the microwave-assisted method could greatly reduce the reaction time, enhancing the production efficiency. Moreover, it can also suppress particle agglomeration effectively, which is conducive to preparing MNMPs with uniform sizes.^83^ With the inherent heating characteristics of microwave, the reaction system achieves more uniform thermal distribution, avoiding localized overheating or undercooling phenomena. Consequently, the prepared MNMPs exhibit greater stability and uniformity.^84^ As shown in Figure 10B, the transmission electron microscopy (TEM) images of MNMPs prepared by the microwave-assisted method clearly demonstrate favorable morphological uniformity and controllable size distribution, from 44 to 162 nm approximately. The MNMPs show a high inherent loss power of 15.21 ± 0.34 nHm^2^/kg in magnetothermal therapy. In photothermal therapy, it can be heated to the therapeutic temperature by 808 nm laser irradiation at a concentration of 100 *μ*g/mL, and has excellent dual-modal hyperthermia performance.^81^ Figure 10C illustrates the morphology and size distribution between 40 and 120 nm of microwave-prepared nanoparticles featuring rod-like or needle-like structures, which facilitate cell adhesion and growth, making them ideal candidates for bone regeneration. This approach produces nanoparticles with uniform dimensions, excellent dispersibility, and bioactive properties, which are suitable for bone regeneration and other biomedical applications.^82^ Furthermore, the microwave-assisted method can often be combined with other methods to further enhance the performance of MNMPs. For example, when combined with the hydrothermal method, MNMPs were mixed with essential oils for antibacterial applications. Studies have found that core-shell structured MNMPs exhibit good crystallinity and magnetic properties, while the loading of essential oils could enhance their antibacterial efficacy significantly.^85^ By combining with the co-precipitation method, MNMPs could be prepared for DNA purification. This approach enables high-quality preparation of nanoparticles with higher efficiency and reproducibility compared to traditional methods.^86^

However, the microwave-assisted method can improve preparation efficiency and inhibit particle agglomeration; it has significant limitations. This method poses great challenges in temperature control, and fluctuations in local thermal distribution are likely to affect the uniformity of the products. Additionally, the required microwave equipment is expensive and consumes large amounts of energy, which restricts large-scale production. Meanwhile, its applicable material range is relatively narrow, as it is only suitable for the preparation of specific magnetic materials such as iron-based oxides and ferrites, making it difficult to meet the demands for diverse functionalities.

#### Spray drying method

**Figure 11:**
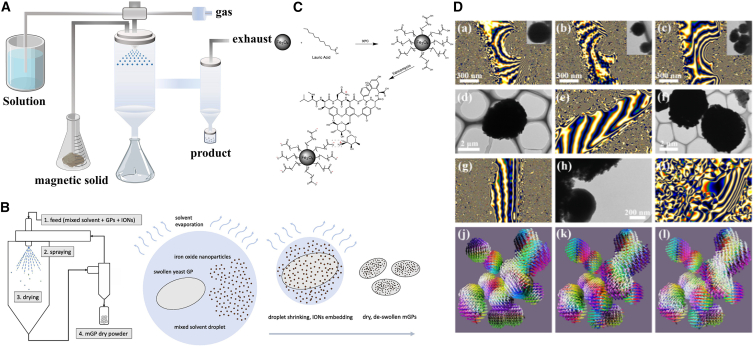
Spray drying method for preparing MNMPs (A) Schematic diagram of the preparation of MNMPs via the spray-drying method. (B) Flowchart of the preparation of magnetic yeast glucan particles using the spray-drying method.^88^ (copyright 2023, ACS). (C) Synthesis scheme of vancomycin-adsorbed MNMPs.^89^ (copyright 2021, Elsevier). (D) Magnetic flux lines and micromagnetic simulation.^90^ (copyright 2020, ACS).

During the spray drying method process, precursors of magnetic materials are mixed, such as metal salts, metal oxides, ferrite precursors or magnetic composite systems, with appropriate additives forming a uniform liquid raw material, which is dispersed into micro-droplets through an atomizer. Under the influence of hot air, these droplets rapidly lose moisture by evaporation, causing the precursors within the droplets to undergo chemical reactions, crystallization, or aggregation. Finally, MNMPs could be formed and collected,^87^ as illustrated in Figure 11A.

In practical applications, MNMPs prepared by the spray drying method have shown great significance for multiple research fields.^91^ In the biomedical field, they can be used to prepare MRI contrast agents and drug carriers,^92^ with excellent dispersibility and adjustable sizes, achieving precise detection and treatment of targeted lesions. Figure 11B shows the procedure of preparing MNMPs by the spray drying method.^88^ Magnetic yeast glucan particles were obtained by dispersing yeast glucan particles and glucan-coated iron oxide nanoparticles in a water-ethanol mixed solvent. Then the solvent was evaporated and spray-dried rapidly, causing the glucan shell to contract and irreversibly encapsulate the nanoparticles inside the particles (approximately 5.1 *μ*m). As shown in Figure 11C, drugs are adsorbed onto the core of MNMPs, followed by spray drying encapsulation into lactose/dextran matrices. This method optimizes aerodynamic performance and enables localizable drug delivery to bacterial pneumonia-infected sites, improving drug loading and delivery efficiency.^89^ The spray drying method enables rapid continuous production with high efficiency, which is suitable for large-scale preparation of MNMPs. By precise adjustment of process parameters, such as atomization settings, hot air temperature, and flow rate, effective control of particle size, morphology, and structure of MNMPs can be achieved (the average size is 1.8 *μ*m, and 90% of the particle diameters are less than 4.3 *μ*m).^93^ Additionally, MNMPs prepared by spray drying exhibit excellent dispersibility, because the drying process could minimize interparticle contact and aggregation opportunities. Figure 11D reveals the magnetic flux line distribution and multi-scale magnetic coupling mechanism of *Fe*_3_*O*_4_ nanospheres in the composite material through electronic holography and micromagnetic simulation, confirming the magnetic flux fusion of adjacent *Fe*_3_*O*_4_ nanospheres and the existence of magnetic coupling effects between microspheres. Under an externally applied alternating magnetic field (AMF), the magnetic moments vibrate synergistically, enhancing the magnetic loss capacity of the material.^90^

However, the spray drying method exhibits significant energy consumption, as the processes of hot air heating and moisture evaporation require substantial energy input, leading to relatively high production costs. For temperature-sensitive magnetic material precursors, excessively high drying temperatures may damage their chemical structures, thereby impairing the magnetic responsiveness of the final products. In addition, this method is highly dependent on atomization parameters, making it prone to problems such as particle agglomeration or excessively wide size distribution, which necessitates additional optimization of process parameters.

#### Sono chemical method

**Figure 12:**
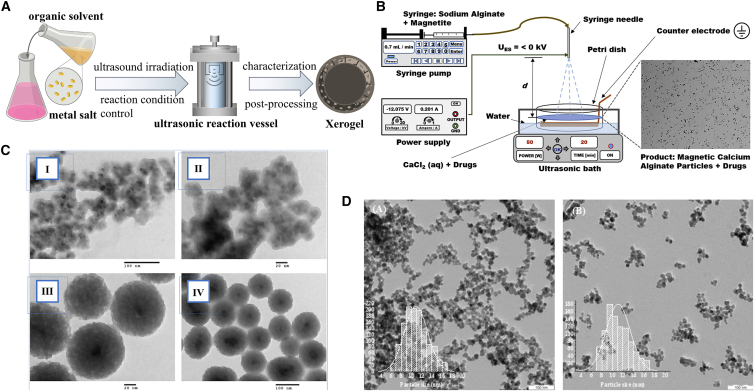
Sono chemical method for preparing MNMPs (A) Schematic diagram of the preparation of MNMPs by the sonochemical method. (B) Schematic diagram of the preparation of MNMPs by the sonochemical method combined with the spray drying method.^95^ (copyright 2018, Elsevier). (C) TEM images of multi-core and single-core MNMPs.^96^ (copyright 2021, Elsevier). (D) TEM images and size distribution histogram of SPIONs.^97^ (copyright 2022, Elsevier).

The sono chemical method, as a unique and efficient approach for preparing MNMPs,^94^ is based on ultrasonic cavitation effect primarily. Materials, such as soluble salts of metals, metal organic compounds or metal oxide precursors, undergo chemical reactions in solvents under the action of ultrasonic waves and form MNMPs. When ultrasound waves act on liquid media, alternating high-pressure and low-pressure cycles are generated. Microscopic bubbles are formed in the low-pressure phase, which rapidly collapse in the high-pressure phase. This process could create instantaneous high temperatures, extreme pressures, and intense shock waves with microjets, as illustrated in Figure 12A. During the preparation, a precursor solution containing magnetic elements is initially prepared. Depending on requirements, surfactants or stabilizers may be added to control particle growth and prevent aggregation. The solution is then treated with ultrasonic waves. Under the action of the ultrasonic cavitation effect, the precursors undergo a series of physical and chemical transformations. The increased temperature and pressure accelerate chemical reactions of metal ions, potentially leading to hydrolysis, redox reactions, and subsequent formation of magnetic material nuclei. The shock waves and microjets provide dynamic energy for the growth of crystal nuclei, which continuously absorb surrounding ions to form MNMPs.^98^

The sono chemical method effectively inhibits particle agglomeration and enhances particle dispersibility and stability, which could be utilized to address the limitations of other methods,^99^ thereby providing new avenues for the preparation of MNMPs. As illustrated in Figure 12B, the Sono Chemical Method is integrated with drying and spraying methods. Vibration from the ultrasonic bath could optimize the formation and characteristics of particles.^95^ Moreover, the instantaneous high temperature and pressure generated by ultrasonic cavitation could significantly accelerate chemical reaction rates, improving preparation efficiency with the size ranging from 20 nm to 10 *μ*m. As shown in Figure 12C, shockwaves generated by ultrasonic-induced bubble collapse prevent the spontaneous aggregation of magnetite nanoparticles to obtain uniform single-core-shell structures and controllable particle sizes (approximately 100 nm). The Ms of MNMPs is approximately 50–62 emu/g. After being coated with chitosan assisted by ultrasound, the saturation magnetization (Ms) can be increased to 86.93 emu/g. In a rotating magnetic field, the movement speed of MNMPs can reach 20 *μ*m/s, and the speed reaches the peak after optimization with the magnetic field frequency, demonstrating efficient magnetic guidance ability.^96^ As illustrated in Figure 12D, TEM images reveal that the prepared MNMPs exhibit standard spherical structures, with relatively uniform particle sizes according to the size distribution histogram inset,^97^ providing excellent performance for biomedical applications. For example, they can function as targeted drug carriers, capable of delivering medications to lesion sites precisely under the guidance of an external magnetic field, thereby enabling highly efficient treatments.^100^

However, the sono-chemical method relies on high-performance ultrasonic equipment, which entails high procurement and maintenance costs.^101^ Moreover, the energy utilization efficiency of the ultrasonic process is relatively low, resulting in energy waste. The reaction mechanism of this method is closely related to the ultrasonic cavitation effect, and the limitation of cavitation regions may lead to non-uniform reactions, thereby impairing the homogeneity of the products.

### Methods comparison

Chapter 2 systematically elaborates on various preparation methods. Each of these methods possesses unique advantages and characteristics, exhibiting distinct strengths in terms of preparation efficiency, product performance, operational adaptability, functional adjustability, and application scalability, as illustrated in Table 4. They thus provide diverse technical options for preparing MNMPs with different requirements.

**Table 4:** Materials and application scenarios of different preparation methods

Method	Preparation cycle	Magnetic material	Application fields
Microwave-assisted	1 to 3 h	Fe_3_O_4_, CoFe_2_O_4_, ZnFe_2_O_4_	targeted tumor drug delivery, magnetic resonance imaging (MRI)
Electrodeposition	4 to 7 h	Ni-Co magnetic films, Au−Fe_3_O_4_	electrode coatings for biomedicine
Emulsification	6 to 10 h	Fe_3_O_4_, MnO_2_ −Fe_3_O_4_	targeted delivery, MRI, cell separation and purification
Spray drying	2 to 4 h	Fe_3_O_4_, Gd_2_O_3_ −Fe_3_O_4_	drug microspheres for release, local drug delivery, magnetic targeted therapy
High temperature	10 to 20 h	Fe_3_O_4_, CoFe_2_O_4_, FePt	tumor magnetic hyperthermia, MRI
Sol-gel	12 to 24 h	Fe_3_O_4_, LaFeO_3_	magnetic targeted therapy, bone regeneration functions
Hydrothermal	10 to 18 h	Fe_3_O_4_, MnFe_2_O_4_, Fe_3_O_4_ −Carbon Quantum	tumor magnetic hyperthermia, MRI, targeted tumor drug delivery
Self-ignition	2 to 5 h	NiFe_2_O_4_, CuFe_2_O_4_	tumor magnetic fluid hyperthermia, bacterial separation and detection
Solvothermal	14 to 24 h	Fe_3_O_4_, CoFe_2_O_4_	targeted delivery, bacterial separation and detection, magnetic hyperthermia therapy
Co-precipitation	2 to 4 h	Fe_3_O_4_, CuFe_2_O_4_	targeted drug delivery, MRI, immune cell sorting, tumor marker detection
Sono chemical	2 to 5 h	Fe_3_O_4_, ZnFe_2_O_4_	tumor magnetic hyperthermia, accelerate drug loading, MRI

Specifically, the microwave-assisted method and sono-chemical method, as auxiliary enhancement techniques, feature high preparation efficiency and can effectively inhibit particle agglomeration, thereby contributing to improved dispersibility of MNMPs. The electrodeposition method has unique advantages in the preparation of magnetic films and ordered structure particles, enabling the directional fabrication of functional magnetic materials. The emulsification method is advantageous for its low cost and can flexibly prepare MNMPs with adjustable sizes according to actual requirements. The spray drying method is suitable for large-scale sample collection and rapid formation of MNMPs, laying a solid foundation for industrial applications. The high-temperature synthesis method can produce MNMPs with high crystallinity, which is beneficial for enhancing the magnetic performance of the final products. The sol-gel method exhibits good compatibility with various materials and can successfully prepare composite MNMPs with uniform components, thereby expanding the functional diversity of MNMPs. Both the hydrothermal method and solvothermal method demonstrate excellent performance in regulating product structures, capable of preparing special-structured MNMPs with high purity and good dispersibility, which are suitable for the fabrication of high-performance MNMPs. The self-ignition method is characterized by fast reaction speed and a simple process, enabling the rapid synthesis of MNMPs within a short period. The co-precipitation method is simple to operate, low in cost, and high in efficiency, making it highly suitable for large-scale preliminary preparation of MNMPs and endowing it with promising industrial application prospects.

**Figure 13:**
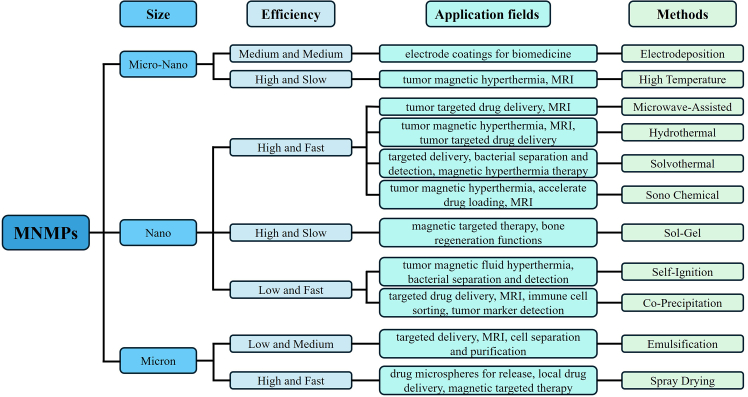
Decision tree for selecting preparation methods based on different factors

Based on the comprehensive comparison of the advantages of various preparation methods mentioned above, the selection of MNMPs preparation methods can be targeted according to actual application needs, performance requirements, and cost budgets, as illustrated in Figure 13. For fields such as biomedicine and catalysis that demand high-purity, uniform-size, and special-structured MNMPs, the hydrothermal method or solvothermal method is an ideal choice, as they can achieve precise regulation of product structure and performance. If rapid and large-scale preparation with low cost is required, the coprecipitation method is preferred, as it can effectively reduce preparation costs while ensuring preparation efficiency. For the preparation of magnetic films or ordered structure particles, the electrodeposition method is recommended to fully exploit its advantages in directional preparation. If it is necessary to improve preparation efficiency and enhance particle dispersibility, the microwave-assisted method or sonochemical method can be used as auxiliary means in combination with other chemical methods to optimize the preparation effect. For scenarios with requirements for environmental protection and functional diversity, the sol-gel method with an optimized solvent system can be selected to realize the preparation of environmentally friendly composite MNMPs. In practical applications, combining two or more preparation methods can give full play to their respective advantages, integrate the strengths of different methods, and further optimize the performance of MNMPs to meet higher application requirements.

#### Biomedical applications of MNMPs

MNMPs have played prominent roles in biomedical applications, owing to their unique physicochemical properties and biocompatibility.^102^ First, their unique magnetic properties allow precise localization under external magnetic field guidance, for instance, enabling the direct delivery of therapeutic agents to lesion sites, which enhances treatment efficacy and minimizes damage to healthy tissues.^103^ Secondly, MNMPs usually exhibit excellent biocompatibility and rarely induce *in vivo* immune responses, which are suitable for *in vivo* diagnostics^104^ and therapeutic applications.^105^ Thirdly, MNMPs are small enough to penetrate biological membranes and interact with biomolecules. During MRI, MNMPs could alter the relaxation time of surrounding water molecules, thereby enhancing imaging contrast and further facilitating early and accurate disease diagnosis.^106^ In addition, MNMPs can assist in cell separation, sorting, and tissue regeneration.^107^ In summary, MNMPs play an irreplaceable and crucial role in biomedical applications due to their distinctive characteristics.

In the biomedical field, the advanced and precise detection^108^ and imaging^109^ of diseases are crucial for effective treatment. Traditional detection and imaging techniques, such as X-rays and ultrasound imaging, face limitations including insufficient resolution and difficulties in detecting small lesions. The usage of MNMPs is a potential solution due to their unique magnetic properties, excellent biocompatibility, and micro/nano size effects. They can be manipulated under external magnetic fields precisely to achieve targeted capture of specific biological units.^110^ Accurate detection of specific biological molecules or cells could be realized with the surface modification of MNMPs.^111^ MNMPs have exhibited extensive application potential in biomedicine, and the subsequent sections will elaborate on their core applications across three key domains: diagnosis, medical imaging, and targeted therapy.

### Diagnosis

**Figure 14:**
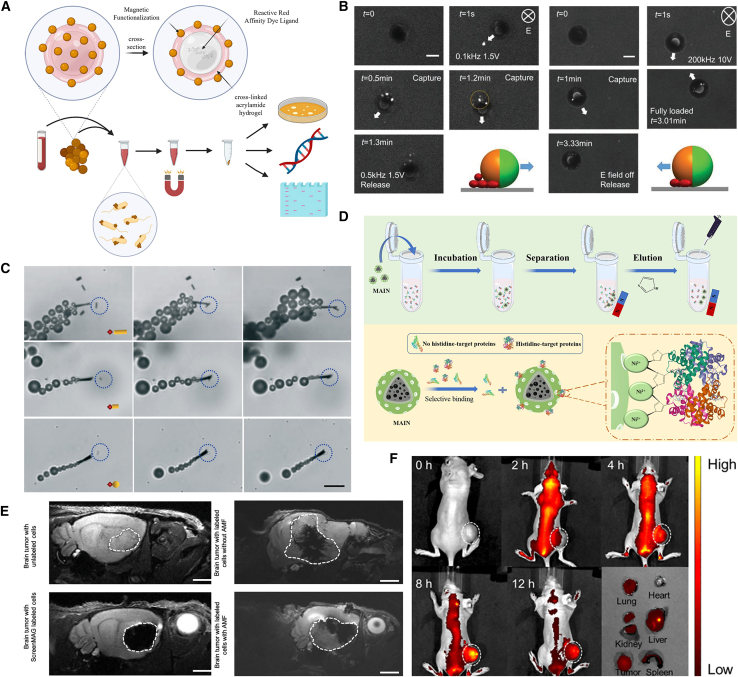
Diagnosis and medical imaging applications of MNMPs (A) Workflow of MNMPs capturing cells.^115^ (copyright 2021, BioMed Central). (B) Process of MNMPs capturing mitochondria.^116^ (copyright 2020, Wiley). (C) Process of MNMPs labeling proteins.^117^ (copyright 2014, Royal Society of Chemistry). (D) Process of MNMPs labeling amino acids and separating proteins.^118^ (copyright 2023, Elsevier). (E) MRI image of mouse upper brain labeled with MNMPs.^119^ (copyright 2021, Springer Nature). (F) Fluorescence image of mouse tumor labeled with MNMPs.^120^ (copyright 2022, Elsevier).

Precise cell isolation is one of the key steps in biomedical research and clinical practice.^112^ As the basic units of life activities, different types of cells play unique roles in physiological and pathological processes.^113^ Due to their distinctive physicochemical properties, MNMPs demonstrate significant potential in cell capture.^114^ As illustrated in Figure 14A, MNMPs enable the capturing and separating of biomarkers in complex biological samples.^115^ They can specifically bind to microbial proteins, nucleic acids, and intact bacterial cells for rapid separation, and the isolated particles are applicable to various diagnostic purposes, such as nucleic acid extraction, protein analysis, and pathogen detection. Notably, magnetic separation enables a detection limit of 10^4^CFU, while increasing the yield of bacterial nucleic acids in whole blood by 4-fold and enriching key virulence factors (e.g., LcrV, F1 antigen) as well as proteins associated with the type III secretion system. Figure 14B demonstrates the process of capturing and transporting mitochondria with MNMPs.^116^ At a frequency of 0.05–0.5 kHz, the dielectric hemispheres are moved forward by induced charge electrophoresis, and mitochondria are injected into the positive dielectrophoresis trap between MNMPs and ITO substrates by induced charge electroosmosis. In contrast, at frequencies of 100–500 kHz, MNMPs are driven forward by self-dielectrophoresis: specifically, at 200 kHz and 10 V, the number of individually trapped mitochondria per trap reaches 4, whereas the trapping efficiency drops to 1 mitochondrion per trap at 14 V—an effect attributed to enhanced hydrodynamic shearing induced by the increased velocity of MNMPs. The selective capture and transport of mitochondria are influenced by the DEP force and particle velocity. This capability provides a new approach for organelle isolation^121^ and analysis.

Because of the complex biofluid environment of the human body and the similar physical properties between cells, it remains challenging to isolate a small number of diseased cells from a large population of normal cells.^122^ MNMPs could address this kind of problem, whose excellent biocompatibility ensures minimal impact on the activity of biomolecules and cells. As illustrated in Figure 14C, MNMPs show versatility and effectiveness for labeling and recognition in a multiplex immunoassay for simultaneous immunoglobulin detection.^117^ Multiple identifications can be achieved by MNMPs specifically binding to target proteins (e.g., immunoglobulins) labeled with gold microstructures of distinct sizes and morphologies, fueled by H_2_O_2_, and relying on the morphological differences of the tags. Notably, MNMPs exhibit a contact-based capture efficiency of 90% for IgG proteins tagged with such size- and shape-diversified gold microstructures, while effectively bypassing unmodified microstructures or non-target proteins. This capability enables label-free visual multiplexed detection without the need for conventional washing steps, simplifying the detection workflow while maintaining high specificity. Figure 14D further depicts the labeling and separation of proteins through MNMPs in complex biological samples.^118^MNMPs specifically bind to histidine residues in proteins via surface-chelated Ni^2+^; the resulting MNMP-target protein complexes are separated by an external magnetic field, and the target protein is subsequently released using an imidazole-containing elution solution to achieve selective separation. This approach exhibits high efficiency and selectivity, as evidenced by key experimental data, MNMPs display a maximum adsorption capacity of 1069.2 mg/g for histidine-rich bovine hemoglobin, can enrich 0.1 mg/mL histidine-tagged RSV-F0 from cell culture medium supernatant, achieve 75% recovery and over 95% purity of BSA when lysozyme is present in 5-fold excess, and retain 97.3% of their adsorption capacity after 9 consecutive cycles. Consequently, MNMPs exhibit immense potential in tumor labeled and separation for biomedical applications, promising transformative advancements in disease diagnosis,^123^ drug development, and cell therapy.

### Medical imaging

As an excellent biomarker, MNMPs are also used in biomedical imaging for disease diagnosis, treatment, and drug development^124^ and have emerged as highly promising new imaging agents.^125^ In addition, surface modification of MNMPs enables them to acquire targeting capabilities, allowing specific binding to specific biomolecules, cells, or tissues for precise imaging of pathological regions. As shown in Figure 14E, MNMPs shorten the transverse relaxation time of protons in surrounding tissues via their high transverse relaxation rate, resulting in low-signal regions in labeled cells or tumors on T_2_-weighted MRI of mouse brains and thus enabling imaging tracking. Notably, magnetic fluid hyperthermia (MFH) under an AMF with a specific absorption rate (SAR) induced 59% mortality in U87-MG cells *in vitro*; *in vivo*, this translated to reduced tumor volumes in mice 42 days post-implantation and an 89% treatment response rate. Concurrently, the T_2_ values of labeled cells increased from 24 ms to 38 ms post-AMF exposure, providing robust data support for both tumor growth monitoring and the therapeutic efficacy of MFH. MRI images of mouse brains show the monitoring of tumor growth and the therapeutic efficacy of MFH.^119^ The tumor regions labeled with MNMPs exhibit distinct dark areas, which delineate tumor boundaries clearly. Figure 14F displays fluorescence images of a mouse tumor labeled with MNMPs,^120^ showing fluorescence intensity changes in tumor regions within 12 h after injection. The magnetic effect of MNMPs could dim the T_2_-weighted MRI signal, and the surface Pt nanoparticles would react with H_2_O_2_ to generate oxygen bubbles to enhance the contrast of US imaging. Meanwhile, the loaded Ce6 generates fluorescence signals under laser excitation, thereby enabling multimodal imaging—specifically, MNMPs exhibit an emission peak at 660 nm upon 480 nm excitation, reach the maximum tumor fluorescence accumulation at 8 h post-injection, possess a T_2_ relaxivity of 128.32 mM^−1^s^−1^ for MRI, and enhance ultrasound imaging signals through Pt-catalyzed O_2_ bubble formation. These experiments confirm the excellent tumor targeting ability and imaging performance of MNMPs, which could effectively mark tumors *in vivo* and provide essential experimental support for subsequent multimodal imaging-guided phototherapy. Therefore, MNMPs could not only enhance diagnostic accuracy in early disease stages but also facilitate personalized treatment strategies.

### Target therapy

In biomedical science, the precision and effectiveness of therapeutic approaches have always been the focused research objectives. However, traditional drug therapies and other treatment methods reveal numerous insurmountable limitations for addressing complex diseases.^126^ MNMPs exhibit excellent magnetic responsiveness, enabling precise guidance to targeted sites under external magnetic fields to achieve the delivery of therapeutic agents.^127^ After being modified with specific recognition molecules, such as antibodies or aptamers, they can be selected to bind to diseased cells and thereby enhance targeting treatment ability.^128^ When serving as carriers for therapeutic substances (e.g., drugs, genes, or cells), MNMPs can protect them from degradation during systemic transport and enhance stability and bioavailability.^129^

**Figure 15:**
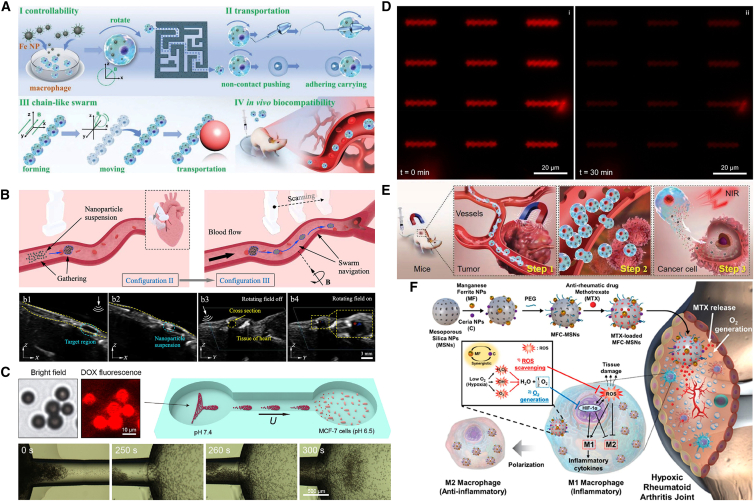
Target therapy applications of MNMPs (A) Schematic illustration and applications of precise trajectory control for MNMPs.^131^ (copyright 2022, Wiley). (B) Transport and navigation of MNMPs in *ex vivo* porcine coronary arteries.^132^ (copyright 2021, AAAS). (C) Self-regulated drug delivery and release capabilities of MNMPs.^133^ (copyright 2023, Wiley). (D) Light triggered drug release from MNMPs.^134^ (copyright 2018, ACS). (E) Schematic diagram of tumor targeted therapy using MNMPs.^135^ (copyright 2021, Wiley). (F) Schematic illustration of the mechanism for cancer therapy employing MNMPs.^136^ (copyright 2022, Elsevier).

The excellent biocompatibility of MNMPs enables them to maintain relative stability within biological systems and minimize adverse reactions. Their magnetic responsiveness enables precise trajectory control under external magnetic field guidance for targeted delivery of therapeutic agents.^130^ As illustrated in Figure 15A, MNMPs are loaded into macrophages to create novel cellular robots.^131^ Through precise control of external rotating magnetic fields, these cellular robots achieve high-precision trajectory navigation in complex environments—featuring a tracking error of only 3.35 μm for rectangular trajectories and 3.73 *μ*m for circular trajectories, the ability to traverse 120 *μ*m-wide micromazes, contact-free movement between cancer cells, and chain-like swarm formation for transporting objects over 100 times their volume. Their magnetic responsiveness and biocompatibility further enable targeted drug release or cell tracking at tumor sites under *in vivo* magnetic field guidance, realizing non-invasive and precise therapy. Figure 15B shows the swarm formation and navigation control of MNMPs in a pig coronary artery *in vitro*.^132^ Ultrasonic Doppler imaging was utilized to capture the red/blue bicolor signals near the microcluster for positioning. The precise navigation of the MNMP population under an external magnetic field can be achieved by reducing blood flow resistance, enhancing anti-interference capability via increased magnetic field frequency, and adopting near-wall navigation in flowing blood—with key performance metrics including a mean velocity of up to 40.8 mm/s under ultrasound Doppler guidance, a nanoparticle access rate exceeding 90%, real-time tracking errors of 0.34 ± 0.19 mm (constant flow) and 0.44 ± 0.21 mm (pulsatile flow), and the ability to form a microswarm within 20 s for *ex vivo* porcine coronary artery navigation. The controllable movement of MNMPs in complex biological environments provides crucial technical foundations for *in vivo* applications of drug delivery.^137^

MNMPs have emerged as ideal materials for constructing novel drug release systems due to their unique physicochemical properties. They can stably exist in physiological environments without inducing severe immune responses or toxicity in organisms. Moreover, through surface modifications, various functional groups or drug molecules can be coupled with MNMPs to create drug carriers.^138^ After reaching the target lesion site, controllable drug release can be achieved with external stimuli such as magnetic fields, temperature, pH, or enzymes. As illustrated in Figure 15C, MNMPs are utilized for dynamic-targeted drug delivery within microfluidic channels.^133^ This method not only enhances drug concentration at tumor sites but also minimizes systemic distribution, thereby reducing toxicity to healthy tissues and providing robust technical support for precision oncology—supported by key performance data: MNMPs exhibit a DOX loading capacity of 180 mg/g, self-organize into swarms with a maximum collective velocity of 180.0 *μ*m/s for targeted delivery over 10 mm under rotating magnetic field control, achieve pH-responsive self-regulated release (53.3% DOX released in 4 h at pH 5.0 vs. 14.2% at pH 7.4), and reduce MCF-7 cell viability to 18.4% at pH 6.5 with negligible cytotoxicity to normal cells. Figure 15D shows that MNMPs are further embedded in microswimmers for light-induced drug release.^134^ Fluorescence microscopic images reveal that the fluorescence intensity decreased significantly in microswimmers following 30 min of light irradiation, indicating successful controllable drug release; 60% of DOX is released within 5 min under 365 nm light at 0.34 W/cm^−2^, with 15% DOX released per minute of irradiation to enable on-demand dosing.

What’s more, the MNMPs can be modified with specific molecules that exhibit high selective binding to the diseased cells, significantly enhancing targeted therapy.^139^ Therapeutic efficacy could be effectively improved by drug enrichment and controlled release at target lesion sites,^140^ while minimizing side effects on healthy tissues. As illustrated in Figure 15E, macrophage-based MNMPs are proposed for targeted tumor therapy *in vivo*.^135^ Guided by a controllable external magnetic field, they can traverse the circulatory system to reach target tumor sites and release drugs under near-infrared (NIR) light stimulation for tumor cell eradication, exhibiting an 85% targeting efficiency in vascular models, achieving a 91% tumor growth inhibition rate following three 8-min irradiations with an 808 nm NIR laser on days 1, 3, and 5, and inducing nearly complete tumor regression by day 7 without significant pathological alterations in major organs. Figure 15F shows the process of driving the MNMPs to tumor regions with applied AMFs for precise drug delivery and localized treatment.^136^ When MNMPs were aggregated in the tumor area through magnetic targeting, they could alleviate hypoxia by catalyzing oxygen production, promote macrophage polarization by regulating reactive oxygen species, and respond to the release of methotrexate to achieve precise combined therapy. To further achieve active targeting, MNMPs can be surface modified with specific ligands to bind to receptors overexpressed on tumor cells.

MNMPs not only hold promising prospects in the biomedical field due to their unique properties, but also have been widely studied in many other fields. In food safety control,^141^ they are used for high-sensitivity quantitative detection^142^ and sensor development.^143^ In the field of environmental science, they serve as efficient adsorbents for pollutant treatment.^144^ Furthermore, they facilitate the development of high-performance catalysts in the energy research area.^145^ Their indispensable role has been established across diverse scientific and industrial fields.^146^

### Challenges of clinical translation

Although MNMPs have exhibited favorable therapeutic efficacy and biocompatibility in preclinical animal models for applications such as tumor magnetic hyperthermia, targeted drug delivery, and MRI contrast enhancement, their translation from animal experiments to human clinical trials is impeded by substantial physiological and pathological disparities between humans and animal models, which give rise to a series of unique challenges in human clinical trials, as illustrated in Table 5. The primary challenge stems from the interspecies differences in biological responses and the complexity of the human *in vivo* microenvironment. The low cytotoxicity and mild immune response observed in small animal models cannot be directly extrapolated to humans, as the human immune system possesses a far more sophisticated regulatory network. Systemically administered MNMPs may therefore elicit unexpected biological reactions, including complement system activation, excessive macrophage phagocytosis, and mild allergic responses. In addition, the human body has a far more complex *in vivo* microenvironment compared with standardized animal models, characterized by heterogeneous blood components, distinct hemodynamic characteristics, and physiological barriers such as vascular endothelial tight junctions and tumor interstitial hypertension. These factors can induce the aggregation of MNMPs in the circulatory system, shorten their *in vivo* circulation time, and severely impede their effective accumulation at target sites, a problem that is negligible in the commonly used subcutaneous xenograft tumor models in preclinical research. Another critical set of challenges in human clinical trials involves human individual heterogeneity, unvalidated long-term biosafety, and the absence of application protocols adapted to human physiological characteristics. Unlike standardized animal models with a unified genetic background and physical status, human subjects exhibit remarkable individual differences in age, physical condition, tumor stage, and hepatic/renal metabolic function, which renders the determination of a universal optimal dosage of MNMPs extremely difficult. Dosages extrapolated from animal body weight and metabolic characteristics may either result in insufficient therapeutic efficacy in some patients or impose potential toxic burdens on others. Furthermore, preclinical animal experiments are mostly short-term studies, leading to a lack of systematic data on the long-term *in vivo* fate of MNMPs in humans, including the metabolic pathways, clearance rates of core magnetic materials, and the potential risk of chronic inflammation or tissue fibrosis induced by the long-term retention of MNMP residues, especially in patients with impaired metabolic function. Moreover, the application protocols validated in animal models are not directly applicable to humans; the penetration depth of external magnetic fields in the human body is inherently limited, and the feasibility and safety of clinical administration routes such as intravenous infusion and intratumoral injection require rigorous verification. For first-in-human trials of MNMPs, there are currently no mature and universal clinical application standards to follow, which further hinders their smooth progression into human clinical research.

**Table 5:** The advantages and limitations of MNMPs in various application fields

Application fields	Advantages	Limitations
MRI	1. Excellent magnetic responsiveness 2. High imaging contrast 3. Partially biodegradable	1. Weak imaging signals in deep tissues 2. Prone to generating artifacts
Magnetic hyperthermia	1. High magnetic-thermal conversion efficiency 2. Can be combined with radiotherapy and chemotherapy	1. Magnetic field penetration depth 2. Prone to damaging normal tissues
Targeted delivery	1. High drug loading rate 2. Low miss rate effect 3. Good biocompatibility	1. Easily engulfed by the body 2. Difficult to penetrate the tumor matrix barrier 3. Good biocompatibility
Biological testing	1. High specificity for detection 2. Little effect on cell damage	1. Non-specific adsorption 2. Magnetic particles are prone to remaining
Bone therapy	1. High adaptability of bone tissue 2. Capable of carrying growth factors and achieving sustained release	1. Poor long-term effect 2. May trigger chronic inflammation

## Conclusion

This review summarizes the fabrication and synthesis strategies of MNMPs as well as their diverse biomedical applications. The physical methods such as emulsification, spray drying, and sono chemical techniques, utilizing mechanical energy or physical fields for preparing high-purity and scalable particles, are environmentally friendly (no toxic reagents, low pollution risk). However, they usually suffer from poor size control and high energy consumption. Chemical methods, including co-precipitation, solvothermal, and hydrothermal techniques, enable precise control of particle size, morphology, and magnetic properties through adjusting precursor ratios, reaction conditions, and surface modification. These methods facilitate surface functionalization to ensure good biocompatibility of prepared MNMPs. However, organic solvents or strong acids/bases are needed during the process, which is difficult to produce directly in industry. The article further explores typical biomedical applications of MNMPs. For image diagnosis, due to their magnetic responsiveness, biocompatibility, and modifiability, targeted signal enhancement, efficient capture of trace biomarkers, and multimodal imaging could be achieved under the guidance of an external magnetic field, significantly enhancing the sensitivity and specificity of disease detection. Moreover, based on their unique structural advantages, MNMPs can not only be used for tumor imaging but also directly kill tumor cells through heat generation by magnetic hyperthermia, achieving a closed-loop strategy of “imaging – treatment.” For therapeutic delivery, targeted drug delivery systems and magnetic hyperthermia therapy based on MNMPs can improve the precision of localized treatment through a controllable external magnetic field and reduce systemic toxicity effectively. Meanwhile, combined with the stimulus-response characteristics such as magnetic field, temperature, and pH, MNMPs can be utilized to trigger drug release at the lesion site. For example, under an AMF, the heat generated by MNMPs causes the polymer shell to rupture, achieving pulsed drug release and enhancing the therapeutic effect.

Nevertheless, MNMPs face significant challenges in translating lab-scale research to clinical applications. For future development, preparation methods could be integrated with multidisciplinary technologies, such as automation and computer vision systems, to enable real-time and precise monitoring of particle size.^147^ Breaking through the limitation of single machine vision monitoring, and integrating multimodal sensing technologies including online Raman spectroscopy, microfluidic nuclear magnetic resonance, and real-time magnetic performance detection, thus achieving comprehensive real-time online monitoring of key performance parameters of MNMPs such as particle size, saturation magnetization, and particle uniformity. Combined with machine learning self-learning models, the intelligent preparation system can not only adjust pump flow rates dynamically according to particle size deviations, but also automatically optimize process parameters in response to batch-to-batch raw material variations, minor equipment deviations, and fluctuations in reaction conditions, thereby ensuring the batch consistency of clinical-grade MNMPs. In addition, easily replaceable modular microfluidic chips can be developed and integrated with robotic arms and automatic liquid dispensing systems to realize full-process automation of MNMP preparation. This intelligent preparation system supports one-click parameter switching, which can flexibly meet the clinical customization requirements for small-batch and multi-specification MNMP products. Concurrently, green solvent systems and low-energy consumption conditions should be developed to address challenges in batch stability and environmental sustainability. For instance, introducing a recyclable solvent system can reduce the discharge of waste liquid. Abandoning traditional chemical iron salts and organic surfactants, microbially synthesized ferritin is adopted as the magnetic core precursor, and mild nucleation reactions driven by microbial catalysis are employed to replace chemical synthesis requiring high temperature and high pressure conditions. Meanwhile, biopolysaccharides and plant-derived surfactants are used to substitute chemical dispersants, thus achieving the full bio-based integration of raw materials, reaction systems, and dispersants throughout the entire preparation process. This green preparation strategy not only drastically reduces energy consumption, but also enhances the biocompatibility of MNMPs and mitigates adverse immune responses associated with their *in vivo* clinical applications.

In biomedical applications, combined with magnetic hyperthermia, drug-controlled release, and multimodal imaging functions, MNMPs can play a more important role in the clinical research of orthopedic diseases such as osteoporosis, joint rehabilitation, and fractures. In addition, the development of biodegradable magnetic materials and bio-inspired targeting modification techniques holds promise for achieving long-term *in vivo* biocompatibility. For example, in patients with chronic diseases such as diabetes, MNMPs can be used to release insulin according to blood sugar concentration for a long period of time. Furthermore, artificial intelligence and organ-on-a-chip technology can be introduced to analyze the dynamic interaction mechanisms between MNMPs and biological systems.

## Acknowledgments

This work was supported in part by the Natural Science Foundation of Xiamen, China under grant no. 3502Z202373059, and in part by the 10.13039/501100003392Natural Science Foundation of Fujian Province under grant nos. 2023J011444, 2023J011445, and in part by the 10.13039/501100021171Guangdong Basic and Applied Basic Research Foundation under grant no. 2021A1515110504.

## Author contributions

Z.H., L.M., W.M., T.L., J.Z., Y.M., J.T., W.S., and W.Z. jointly wrote this review article.

## Declaration of interests

There are no conflicts to declare.

## Declaration of generative AI and AI-assisted technologies in the writing process

During the process of writing the manuscript, the artificial intelligence models we used were: Gemini, DeepSeek, and ChatGPT4.0. These generative artificial intelligence and AI-assisted technologies were only employed for non-substantive auxiliary tasks, including but not limited to language polishing, grammar and spelling correction, optimization of sentence logical coherence, adjustment of reference citation format, and improvement of expression clarity. It is important to note that these technologies were not used to generate any core content of the manuscript, such as research hypotheses, experimental designs, data analysis results, conclusions, or key discussions related to the core of the research.
